# Supplementary material for: Dynalign II: common secondary structure prediction for RNA homologs with domain insertions
Source: Nucleic Acids Res. 2014 Nov 21;42(22):13939–48. doi: 10.1093/nar/gku1172 (PMC4267632; doi:10.1093/nar/gku1172)
Supplement: SUPPLEMENTARY DATA [file supp_gku1172_nar-02021-z-2014-File012.zip › manual/javadoc/RNAstructure_java_interface/source/windows/TurboFoldWindow.html]

TurboFoldWindow


JavaScript is disabled on your browser.


- Overview
- Package
- Class
- Tree
- Index
- Help

- Prev Class
- Next Class

- Frames
- No Frames

- All Classes

- Summary:
- Nested |
- Field |
- Constr |
- Method

- Detail:
- Field |
- Constr |
- Method


RNAstructure\_java\_interface.source.windows

## Class TurboFoldWindow

- java.lang.Object
- - java.awt.Component
  - - java.awt.Container
    - - javax.swing.JComponent
      - - javax.swing.JInternalFrame
        - - RNAstructure\_java\_interface.source.windows.InternalWindow
          - - RNAstructure\_java\_interface.source.windows.ModuleWindow
            - - RNAstructure\_java\_interface.source.windows.MultiWindow
              - - RNAstructure\_java\_interface.source.windows.TurboFoldWindow

- All Implemented Interfaces:
  :   ActionListener, ImageObserver, MenuContainer, Serializable, EventListener, Accessible, RootPaneContainer, WindowConstants

  ---

    

  ```
  public class TurboFoldWindow
  extends MultiWindow
  ```

  A class responsible for initializing and running the TurboFold module,
  which calculates common secondary structures of multiple sequences using
  the partition function and probabalistic alignments. This module is only
  used with RNA.

  See Also:
  :   Serialized Form

- - ### Nested Class Summary

    - ### Nested classes/interfaces inherited from class javax.swing.JInternalFrame

      `JInternalFrame.AccessibleJInternalFrame, JInternalFrame.JDesktopIcon`
    - ### Nested classes/interfaces inherited from class javax.swing.JComponent

      `JComponent.AccessibleJComponent`
    - ### Nested classes/interfaces inherited from class java.awt.Container

      `Container.AccessibleAWTContainer`
    - ### Nested classes/interfaces inherited from class java.awt.Component

      `Component.AccessibleAWTComponent, Component.BaselineResizeBehavior, Component.BltBufferStrategy, Component.FlipBufferStrategy`
  - ### Field Summary

    Fields

    | Modifier and Type | Field and Description |
    | --- | --- |
    | `private CardLayout` | `cards` The layout that holds the dynamic options applicable to a mode. |
    | `private static String[]` | `modeNames` An array that holds the names of the TurboFold modes. |
    | `private static long` | `serialVersionUID` |
    | `private JPanel` | `specificPanel` The panel that holds the dynamic options applicable to a mode. |

    - ### Fields inherited from class RNAstructure\_java\_interface.source.windows.MultiWindow

      `columnWidth, imageDialogs, options`
    - ### Fields inherited from class RNAstructure\_java\_interface.source.windows.ModuleWindow

      `isRNA, progressBar`
    - ### Fields inherited from class RNAstructure\_java\_interface.source.windows.InternalWindow

      `backend, dialogHandler, menuBar`
    - ### Fields inherited from class javax.swing.JInternalFrame

      `closable, CONTENT_PANE_PROPERTY, desktopIcon, FRAME_ICON_PROPERTY, frameIcon, GLASS_PANE_PROPERTY, iconable, IS_CLOSED_PROPERTY, IS_ICON_PROPERTY, IS_MAXIMUM_PROPERTY, IS_SELECTED_PROPERTY, isClosed, isIcon, isMaximum, isSelected, LAYERED_PANE_PROPERTY, maximizable, MENU_BAR_PROPERTY, resizable, ROOT_PANE_PROPERTY, rootPane, rootPaneCheckingEnabled, title, TITLE_PROPERTY`
    - ### Fields inherited from class javax.swing.JComponent

      `accessibleContext, listenerList, TOOL_TIP_TEXT_KEY, ui, UNDEFINED_CONDITION, WHEN_ANCESTOR_OF_FOCUSED_COMPONENT, WHEN_FOCUSED, WHEN_IN_FOCUSED_WINDOW`
    - ### Fields inherited from class java.awt.Component

      `BOTTOM_ALIGNMENT, CENTER_ALIGNMENT, LEFT_ALIGNMENT, RIGHT_ALIGNMENT, TOP_ALIGNMENT`
    - ### Fields inherited from interface javax.swing.WindowConstants

      `DISPOSE_ON_CLOSE, DO_NOTHING_ON_CLOSE, EXIT_ON_CLOSE, HIDE_ON_CLOSE`
    - ### Fields inherited from interface java.awt.image.ImageObserver

      `ABORT, ALLBITS, ERROR, FRAMEBITS, HEIGHT, PROPERTIES, SOMEBITS, WIDTH`
  - ### Constructor Summary

    Constructors

    Constructor and Description

    `TurboFoldWindow()`

    Constructor.
  - ### Method Summary

    Methods

    | Modifier and Type | Method and Description |
    | --- | --- |
    | `protected void` | `activate()` Activate data input for this window. |
    | `protected void` | `addBackendTuple(String seq, String ct)` Add a tuple to the calculation back end. |
    | `protected void` | `buildSpecificOptionsPanel()` Build an input options box specific to this window. |
    | `protected void` | `deleteBackendTuple(int index)` Delete a tuple from the calculation back end. |
    | `protected void` | `executeModuleAction(String command)` Execute an action within a particular module. |
    | `protected int` | `getNumSequences()` Get the number of sequences this window handles. |
    | `protected String` | `getSequenceSetAsString()` Get a representation of the sequence set as a string. |
    | `protected void` | `runMainCalculationMultipleSequences()` Run the multiple sequence dependent module calculation in the back end. |

    - ### Methods inherited from class RNAstructure\_java\_interface.source.windows.MultiWindow

      `makeInputControls, runMainCalculation, setMenus, showImageDialogs`
    - ### Methods inherited from class RNAstructure\_java\_interface.source.windows.ModuleWindow

      `combineFileNames, doActions, finishModule, getInputControl, getInputControl, makeStartButton, moduleInitialized, placeComponent, replaceExtension, setAnchorCenter, setAnchorNorth, setFillCenter, setFillHorizontal, setGrid, setInsets, setPad, showProgressBarDeterminate, showProgressBarIndeterminate`
    - ### Methods inherited from class RNAstructure\_java\_interface.source.windows.InternalWindow

      `actionPerformed, setTitles, viewWindow`
    - ### Methods inherited from class javax.swing.JInternalFrame

      `addImpl, addInternalFrameListener, createRootPane, dispose, doDefaultCloseAction, fireInternalFrameEvent, getAccessibleContext, getContentPane, getDefaultCloseOperation, getDesktopIcon, getDesktopPane, getFocusCycleRootAncestor, getFocusOwner, getFrameIcon, getGlassPane, getInternalFrameListeners, getJMenuBar, getLastCursor, getLayer, getLayeredPane, getMenuBar, getMostRecentFocusOwner, getNormalBounds, getRootPane, getTitle, getUI, getUIClassID, getWarningString, hide, isClosable, isClosed, isFocusCycleRoot, isIcon, isIconifiable, isMaximizable, isMaximum, isResizable, isRootPaneCheckingEnabled, isSelected, moveToBack, moveToFront, pack, paintComponent, paramString, remove, removeInternalFrameListener, reshape, restoreSubcomponentFocus, setClosable, setClosed, setContentPane, setCursor, setDefaultCloseOperation, setDesktopIcon, setFocusCycleRoot, setFrameIcon, setGlassPane, setIcon, setIconifiable, setJMenuBar, setLayer, setLayer, setLayeredPane, setLayout, setMaximizable, setMaximum, setMenuBar, setNormalBounds, setResizable, setRootPane, setRootPaneCheckingEnabled, setSelected, setTitle, setUI, show, toBack, toFront, updateUI`
    - ### Methods inherited from class javax.swing.JComponent

      `addAncestorListener, addNotify, addVetoableChangeListener, computeVisibleRect, contains, createToolTip, disable, enable, firePropertyChange, firePropertyChange, firePropertyChange, fireVetoableChange, getActionForKeyStroke, getActionMap, getAlignmentX, getAlignmentY, getAncestorListeners, getAutoscrolls, getBaseline, getBaselineResizeBehavior, getBorder, getBounds, getClientProperty, getComponentGraphics, getComponentPopupMenu, getConditionForKeyStroke, getDebugGraphicsOptions, getDefaultLocale, getFontMetrics, getGraphics, getHeight, getInheritsPopupMenu, getInputMap, getInputMap, getInputVerifier, getInsets, getInsets, getListeners, getLocation, getMaximumSize, getMinimumSize, getNextFocusableComponent, getPopupLocation, getPreferredSize, getRegisteredKeyStrokes, getSize, getToolTipLocation, getToolTipText, getToolTipText, getTopLevelAncestor, getTransferHandler, getVerifyInputWhenFocusTarget, getVetoableChangeListeners, getVisibleRect, getWidth, getX, getY, grabFocus, isDoubleBuffered, isLightweightComponent, isManagingFocus, isOpaque, isOptimizedDrawingEnabled, isPaintingForPrint, isPaintingOrigin, isPaintingTile, isRequestFocusEnabled, isValidateRoot, paint, paintBorder, paintChildren, paintImmediately, paintImmediately, print, printAll, printBorder, printChildren, printComponent, processComponentKeyEvent, processKeyBinding, processKeyEvent, processMouseEvent, processMouseMotionEvent, putClientProperty, registerKeyboardAction, registerKeyboardAction, removeAncestorListener, removeNotify, removeVetoableChangeListener, repaint, repaint, requestDefaultFocus, requestFocus, requestFocus, requestFocusInWindow, requestFocusInWindow, resetKeyboardActions, revalidate, scrollRectToVisible, setActionMap, setAlignmentX, setAlignmentY, setAutoscrolls, setBackground, setBorder, setComponentPopupMenu, setDebugGraphicsOptions, setDefaultLocale, setDoubleBuffered, setEnabled, setFocusTraversalKeys, setFont, setForeground, setInheritsPopupMenu, setInputMap, setInputVerifier, setMaximumSize, setMinimumSize, setNextFocusableComponent, setOpaque, setPreferredSize, setRequestFocusEnabled, setToolTipText, setTransferHandler, setUI, setVerifyInputWhenFocusTarget, setVisible, unregisterKeyboardAction, update`
    - ### Methods inherited from class java.awt.Container

      `add, add, add, add, add, addContainerListener, addPropertyChangeListener, addPropertyChangeListener, applyComponentOrientation, areFocusTraversalKeysSet, countComponents, deliverEvent, doLayout, findComponentAt, findComponentAt, getComponent, getComponentAt, getComponentAt, getComponentCount, getComponents, getComponentZOrder, getContainerListeners, getFocusTraversalKeys, getFocusTraversalPolicy, getLayout, getMousePosition, insets, invalidate, isAncestorOf, isFocusCycleRoot, isFocusTraversalPolicyProvider, isFocusTraversalPolicySet, layout, list, list, locate, minimumSize, paintComponents, preferredSize, printComponents, processContainerEvent, processEvent, remove, removeAll, removeContainerListener, setComponentZOrder, setFocusTraversalPolicy, setFocusTraversalPolicyProvider, transferFocusDownCycle, validate, validateTree`
    - ### Methods inherited from class java.awt.Component

      `action, add, addComponentListener, addFocusListener, addHierarchyBoundsListener, addHierarchyListener, addInputMethodListener, addKeyListener, addMouseListener, addMouseMotionListener, addMouseWheelListener, bounds, checkImage, checkImage, coalesceEvents, contains, createImage, createImage, createVolatileImage, createVolatileImage, disableEvents, dispatchEvent, enable, enableEvents, enableInputMethods, firePropertyChange, firePropertyChange, firePropertyChange, firePropertyChange, firePropertyChange, firePropertyChange, getBackground, getBounds, getColorModel, getComponentListeners, getComponentOrientation, getCursor, getDropTarget, getFocusListeners, getFocusTraversalKeysEnabled, getFont, getForeground, getGraphicsConfiguration, getHierarchyBoundsListeners, getHierarchyListeners, getIgnoreRepaint, getInputContext, getInputMethodListeners, getInputMethodRequests, getKeyListeners, getLocale, getLocation, getLocationOnScreen, getMouseListeners, getMouseMotionListeners, getMousePosition, getMouseWheelListeners, getName, getParent, getPeer, getPropertyChangeListeners, getPropertyChangeListeners, getSize, getToolkit, getTreeLock, gotFocus, handleEvent, hasFocus, imageUpdate, inside, isBackgroundSet, isCursorSet, isDisplayable, isEnabled, isFocusable, isFocusOwner, isFocusTraversable, isFontSet, isForegroundSet, isLightweight, isMaximumSizeSet, isMinimumSizeSet, isPreferredSizeSet, isShowing, isValid, isVisible, keyDown, keyUp, list, list, list, location, lostFocus, mouseDown, mouseDrag, mouseEnter, mouseExit, mouseMove, mouseUp, move, nextFocus, paintAll, postEvent, prepareImage, prepareImage, processComponentEvent, processFocusEvent, processHierarchyBoundsEvent, processHierarchyEvent, processInputMethodEvent, processMouseWheelEvent, remove, removeComponentListener, removeFocusListener, removeHierarchyBoundsListener, removeHierarchyListener, removeInputMethodListener, removeKeyListener, removeMouseListener, removeMouseMotionListener, removeMouseWheelListener, removePropertyChangeListener, removePropertyChangeListener, repaint, repaint, repaint, resize, resize, setBounds, setBounds, setComponentOrientation, setDropTarget, setFocusable, setFocusTraversalKeysEnabled, setIgnoreRepaint, setLocale, setLocation, setLocation, setName, setSize, setSize, show, size, toString, transferFocus, transferFocusBackward, transferFocusUpCycle`
    - ### Methods inherited from class java.lang.Object

      `clone, equals, finalize, getClass, hashCode, notify, notifyAll, wait, wait, wait`

- - ### Field Detail


    - #### cards

      ```
      private CardLayout cards
      ```

      The layout that holds the dynamic options applicable to a mode.


    - #### modeNames

      ```
      private static final String[] modeNames
      ```

      An array that holds the names of the TurboFold modes.


    - #### serialVersionUID

      ```
      private static final long serialVersionUID
      ```

      See Also:
      :   Constant Field Values


    - #### specificPanel

      ```
      private JPanel specificPanel
      ```

      The panel that holds the dynamic options applicable to a mode.
  - ### Constructor Detail


    - #### TurboFoldWindow

      ```
      public TurboFoldWindow()
      ```

      Constructor.
        
        
      Note that since this module is unique to RNA, the nucleic acid type is
      hard-coded as RNA.
  - ### Method Detail


    - #### activate

      ```
      protected void activate()
      ```

      **Description copied from class: `MultiWindow`**

      Activate data input for this window.

      **Specified by:**
      :   `activate` in class `MultiWindow`


    - #### addBackendTuple

      ```
      protected void addBackendTuple(String seq,
                         String ct)
      ```

      **Description copied from class: `MultiWindow`**

      Add a tuple to the calculation back end.

      **Specified by:**
      :   `addBackendTuple` in class `MultiWindow`

      Parameters:
      :   `seq` - The sequence file.
      :   `ct` - The ct file.


    - #### buildSpecificOptionsPanel

      ```
      protected void buildSpecificOptionsPanel()
      ```

      **Description copied from class: `MultiWindow`**

      Build an input options box specific to this window.

      **Specified by:**
      :   `buildSpecificOptionsPanel` in class `MultiWindow`


    - #### deleteBackendTuple

      ```
      protected void deleteBackendTuple(int index)
      ```

      **Description copied from class: `MultiWindow`**

      Delete a tuple from the calculation back end.

      **Specified by:**
      :   `deleteBackendTuple` in class `MultiWindow`

      Parameters:
      :   `index` - The index of the tuple to delete.


    - #### executeModuleAction

      ```
      protected void executeModuleAction(String command)
      ```

      **Description copied from class: `ModuleWindow`**

      Execute an action within a particular module.

      **Overrides:**
      :   `executeModuleAction` in class `MultiWindow`

      Parameters:
      :   `command` - The command that signifies a particular action.


    - #### getNumSequences

      ```
      protected int getNumSequences()
      ```

      **Description copied from class: `MultiWindow`**

      Get the number of sequences this window handles.

      **Specified by:**
      :   `getNumSequences` in class `MultiWindow`

      Returns:
      :   The number of sequences this window handles.


    - #### getSequenceSetAsString

      ```
      protected String getSequenceSetAsString()
      ```

      **Description copied from class: `MultiWindow`**

      Get a representation of the sequence set as a string.

      **Specified by:**
      :   `getSequenceSetAsString` in class `MultiWindow`

      Returns:
      :   The sequence set as a string.


    - #### runMainCalculationMultipleSequences

      ```
      protected void runMainCalculationMultipleSequences()
      ```

      **Description copied from class: `MultiWindow`**

      Run the multiple sequence dependent module calculation in the back end.

      **Specified by:**
      :   `runMainCalculationMultipleSequences` in class `MultiWindow`


- Overview
- Package
- Class
- Tree
- Index
- Help

- Prev Class
- Next Class

- Frames
- No Frames

- All Classes

- Summary:
- Nested |
- Field |
- Constr |
- Method

- Detail:
- Field |
- Constr |
- Method
